# Supplementary material for: Explaining COVID-19 mortality among immigrants in Sweden from a social determinants of health perspective (COVIS): protocol for a national register-based observational study
Source: BMJ Open. 2023 Apr 24;13(4):e070670. doi: 10.1136/bmjopen-2022-070670 (PMC10151235; doi:10.1136/bmjopen-2022-070670)
Supplement: Author's manuscript [file bmjopen-2022-070670.draft_revisions.pdf]

BMJ Open is committed to open peer review. As part of this commitment we make the peer review history of every article we publish publicly available.

When an article is published we post the peer reviewers' comments and the authors' responses online. We also post the versions of the paper that were used during peer review. These are the versions that the peer review comments apply to.

The versions of the paper that follow are the versions that were submitted during the peer review process. They are not the versions of record or the final published versions. They should not be cited or distributed as the published version of this manuscript.

BMJ Open is an open access journal and the full, final, typeset and author-corrected version of record of the manuscript is available on our site with no access controls, subscription charges or pay-per-view fees (http://bmjopen.bmj.com).

If you have any questions on BMJ Open's open peer review process please email info.bmjopen@bmj.com

BMJ Open

Explaining COVID-19 mortality among immigrants in Sweden from a social determinant of health perspective (COVIS). Protocol for a population-register study

Journal:	*BMJ Open*
Manuscript ID	bmjopen-2022-070670
Article Type:	Protocol
Date Submitted by the Author:	30-Nov-2022

Complete List of Authors:	Juárez , Sol Pia; Stockholm University, Department of Public Health Sciences; Centre for Health Equity Studies, Stockholm University/Karolinska Institute<br>Honkaniemi, Helena ; Stockholm University,<br>Aradhya, Siddartha ; Stockholm University,<br>Debiasi, Enrico; Stockholms Universitet, Department of Public Health Sciences<br>Katikireddi, Srinivasa; University of Glasgow, MRC/CSO Social & Public Health Sciences Unit<br>Mussino, Eleonora; Stockholms Universitet, Stockholm University Demography Unit<br>Rostila, Mikael ; Stockholm University, Department of Public Health Sciences

Keywords:	COVID-19, INFECTIOUS DISEASES, SOCIAL MEDICINE

SCHOLARONE™
Manuscripts

Explaining COVID-19 mortality among immigrants in Sweden from a social determinant of health perspective (COVIS). Protocol for a population-register study

Sol P. Juárez[1,2*] sol.juarez@su.se, ORCID iD: 0000-0001-9086-7588

Helena Honkaniemi[1,2] helena.honkaniemi@su.se, ORCID iD: 0000-0003-0800-0892

Siddartha Aradhya[3] siddartha.aradhya@sociology.su.se, ORCID iD: 0000-0003-3748-6270

Enrico Debiasi[1,2] enrico.debiasi@su.se, ORCID iD:0000-0001-5772-5510

Srinivasa Vittal Katikireddi[4] vittal.katikireddi@glasgow.ac.uk, ORCID iD: 0000-0001-6593-9092

Eleonora Mussino[3] eleonora.mussino@sociology.su.se, ORCID iD: 0000-0002-5311-4277

Mikael Rostila[1,2] mikael.rostila@su.se, ORCID iD: 0000-0002-6973-0381

[1] Department of Public Health Sciences, Stockholm University, Stockholm, Sweden.

[2] Centre for Health Equity Studies, Stockholm University/Karolinska Institutet, Stockholm, Sweden.

[3] Demographic Unit (SUDA), Department of Sociology, Stockholm University, Stockholm, Sweden.

[4] Institute of Health & Wellbeing. University of Glasgow

[*] Corresponding author: Sol P. Juárez, Department of Public Health Sciences, Stockholm University, SE-106 91, Stockholm, Sweden

Word count: 3762 (up to 4000)
Abstract: 260 (up to 300)
Illustrations: 1
References: 30

For peer review only

Abstract

Introduction: Adopting a social determinant of health perspective, this project aims to study the extent to which disproportionate COVID-19 mortality among immigrants in Sweden is explained by social factors operating through *differential exposure* to the virus (e.g., by being more likely to work in high-exposure occupations) and *differential effects* of infection arising from socially-patterned, pre-existing health conditions, differential healthcare seeking and inequitable healthcare provision.

Methods and analysis: This study will use health (e.g. hospitalizations, deaths) and sociodemographic information (e.g., occupation, income, social benefits) from Swedish national registers linked using unique personal identity numbers. The study population includes all adults registered in Sweden in the year before the start of the pandemic, as well as individuals who immigrated to Sweden or turned 18 years of age after the start of the pandemic. Our analyses will primarily cover the period from January 31st, 2020 until December 31st, 2022, with updates depending on the progression of the pandemic. We will first evaluate excess COVID-19 mortality between foreign- and Swedish-born individuals examining each mechanism (differential exposure and effects) separately, while considering the potential effect modification between country of birth and socioeconomic factors. Second, we will examine the different mechanisms simultaneously using different advanced statistical modelling techniques, such as mediation analysis and three-way decomposition approach.

Ethics and dissemination: This project has been granted all necessary ethical permissions from the National Ethical Review Board (Dnr 2022-0048-01) for accessing and analysing de-identified data. The final outputs will primarily be disseminated as scientific articles published in open-access peer-reviewed international journals, as well as press releases and policy briefs.

For peer review only

STRENGTHS AND LIMITATIONS OF THIS STUDY

- The study will offer a thorough empirical evaluation of how social conditions shape group risks in the context of a pandemic giving rise to native-immigrant inequalities in COVID-19 mortality.
- The study will offer a comprehensive understanding of native-immigrant inequalities across the COVID-19 disease pathway (i.e., positive test, hospitalization, intensive care unit admission and death).
- This study will be restricted to social information available in administrative registers, therefore it will not be able to account for self-reported racism and discrimination.
- Analyses will not be able to include undocumented immigrants, a highly vulnerable population.

For peer review only

INTRODUCTION

The severe acute respiratory syndrome coronavirus 2 (SARS-CoV-2) and its related disease (COVID-19) represent an unprecedented public health concern, with 612 million cases and 6.5 million deaths internationally as of late September 2022.[1] Since the early stages of the pandemic, immigrants and ethnic minorities have been disproportionately affected by COVID-19. Notably, evidence from the UK and US has found that ethnic minorities are not only at a higher risk of being infected by SARS-CoV-2, but also of developing severe complications and dying from COVID-19, relative to majority ethnic groups.[2-5] A systematic review and meta-analysis has confirmed this pattern for immigrants in most high-income countries.[6]

Sweden has been no exception. Despite its generous immigrant integration approaches and guarantee of equal access to healthcare and social protection for all documented immigrants, immigrants from low- to high-income countries have exhibited excess COVID-19 mortality in Sweden.[7] However, some notable differences have been found by immigrants' region of origin. For example, while immigrants from Finland and Chile have shown moderately higher COVID-19 mortality risks than native-born Swedes, immigrants from other origins show relative risks approximately two (e.g., Iran, Iraq), five (e.g., Syria, Lebanon), and eight times (e.g., Somalia) higher than that of natives.[7] This observation contrasts sharply with the mortality advantage seen among immigrants relative to native Swedes for most other causes, both before and during the pandemic.[7,9-11]

In the international literature, multiple hypotheses have been put forth to explain the excess COVID-19 mortality observed among immigrants and ethnic minorities, two categories which are commonly examined together. In the early months of the pandemic, explanations often focused on biological arguments (including the role of genetic predispositions and vitamin D levels), with politicians claiming that the 'virus does not discriminate' by social background.[12] However, as the pandemic continued, hypotheses involving social factors gained more attention, especially as the empirical evidence began to contradict previous reductive biological arguments. For one, excess COVID-19 mortality has been observed across a wide range of ethnic minority groups, calling into question the role of unique biological drivers.[5] Furthermore, COVID-19 has been found to have a disproportionately greater impact on lower socioeconomic groups,[13,14] illustrating the socially unequal effects of the pandemic.

While the research and political debate regarding the disproportionate impact of the pandemic on minorities is now generally discussed within a broader framework of social inequalities, empirical research on the *mechanisms* behind the observed inequalities is limited. A mechanistic understanding of the role of social inequalities in the pandemic is crucial for addressing the effects of the current crisis on already disadvantaged immigrant and ethnic minority groups, while equipping the public health field for potential future outbreaks.

Theoretical foundation: Social Determinants of Health (SDH)

This study is rooted in the *Social Determinants of Health* (SDH),[15] a theoretical framework that considers health to be a product of various social factors operating at different levels (individual, family, community and/or society) giving rise to social distributions of health and disease in the population. Although broad, the SDH framework lays the foundation for the study of health inequalities, focusing on the underlying social causes of manifested biological differences between groups.

The SDH framework becomes more informative in conjunction with other theoretical perspectives. For example, researchers should also account for the Developmental Origins of Health and Disease (DOHaD)[16] and the life-course[17] perspectives on health, which recognize that poor health is not only the result of the immediate social circumstances of an individual or group, but also of the conditions that are present at birth or that accumulate over the lifespan. Within these frameworks, health inequalities can be conceptualized as the biological expression of unequal lives, which can persist within and across generations.

The above-mentioned frameworks lay the foundation for understanding how health inequalities are (re)produced by social factors operating through exposure and susceptibility.[18] This collective theory of health inequalities has been recently adapted to the context of the COVID-19 pandemic, both for the Swedish general population[13,19] and for ethnic minority groups internationally.[20] In this study, we will further adapt the theory to specifically consider inequalities in COVID-19 mortality among immigrants in Sweden.

Our theoretical expectation is that inequalities in COVID-19 mortality could arise from social factors operating through various points in the disease pathway (**Figure 1**). First, social factors may result in *differential exposure* to the virus, i.e., modifying the risk of being infected with SARS-CoV-2 in the first place. Social factors may also influence the development of the disease once the infection has occurred, with *differential effects* due to *disease susceptibility* (i.e., socially patterned pre-existing health), as well as *individual* (i.e., healthcare seeking) and *structural* (i.e., healthcare provision) *responses*. These mechanisms, independently or in combination with one another, may therefore lead to differential risks of COVID-19 mortality.

More specifically, *differential exposure* to the virus[13,20] refers to how social conditions can place immigrants at greater risk of being infected by SARS-CoV-2. For example, immigrants may be at risk by participating in frontline jobs or living in densely populated or economically-deprived areas with fewer opportunities to maintain a safe physical distance (e.g., in neighborhoods with small local stores and recreational facilities, fewer transport options for commuting, less access to outdoor space, etc.).

Differential effects via *disease susceptibility*[13,19] (also referred to as *vulnerability*[20]) relates to the biological capacity to fight against the virus once the infection has taken place. Immigrants may be at greater risk of mortality due to COVID-19 if they have more pre-existing health conditions or comorbidities, poorer nutritional status or immune response than native Swedes.[20] The concept of susceptibility has traditionally been used

For peer review only

to refer to a genetic predisposition to certain diseases, which immediately removes from the debate the topic of inequalities (or inequities, i.e., unfair and avoidable differences) to focus on *fixed* group-level variations. However, recent developments in epigenetics highlight the role of environmental conditions (including social inequalities) in shaping the expression of our genes and predisposing certain individuals and groups to poor health.[21] Such research allows us to situate the concept of susceptibility in the context of social inequalities.

Inequalities might also arise from *differential effects* of infection via *individual and structural responses*, that is, the inclination and capacity to seek and provide necessary healthcare. *Individual* inclination to seek care may depend on one's trust in the healthcare system (depending on e.g., prior experiences of discrimination), while one's capacity may be dependent on the knowledge required to navigate the system (depending on e.g., language proficiency and health literacy). Furthermore, individual responses can to a large extent be conditioned by *structural* responses, or the capacity of the healthcare system to adapt to the demands of the pandemic while maintaining an equitable response across all social groups. Unlike disease susceptibility, which would be expected to remain stable throughout the course of the pandemic, structural response capacity may evolve and improve over time as the healthcare system adapts to the pandemic and more long-term solutions are introduced, such as the vaccination campaign.

The above-mentioned mechanisms are not mutually exclusive.[20] Most adverse social factors are likely to have a joint effect on COVID-19 outcomes by operating across multiple mechanisms, not only resulting in an additive response on health but a multiplicative burden. For example, a greater risk of *exposure* to the virus in certain occupations might be exacerbated among individuals with high disease susceptibility who experience delayed or inadequate access to health care (i.e., *differential effects*), resulting in more severe COVID-19 outcomes. In fact, given that social inequalities are implicated in all these mechanisms, it is actually expected that *differential exposure* to the virus and *differential effects* of infection would be highly correlated.

Structural racism can also play a differential role in disease prevention and treatment across groups, and can be concealed by, for example, public health actions (i.e., *differential effectiveness of control measures*)[20] influencing all the above-mentioned mechanisms. Although we do not aim to estimate the role of structural racism in this study, we will take it into consideration when discussing the results

.

Aim/objectives and research questions (RQ)

The purpose of this project is to study the extent to which excess COVID-19 mortality among immigrants in Sweden is explained by social determinants acting through *differential exposure* to infection (e.g., by being more likely to work in high-exposure occupations) and by *differential effects* of infection resulting from socially-patterned health conditions (i.e., *disease susceptibility*) as well as social discrepancies in individual healthcare seeking and/or structural provision of healthcare (i.e., *individual or structural response*).

For peer review only - http://bmjopen.bmj.com/site/about/guidelines.xhtml

RQ1: Are immigrants more likely to be exposed to SARS-CoV-2 infection than natives (*differential exposure to the virus*)?

RQ2: Are immigrants more likely to have socially-patterned health conditions that predispose them to developing more severe versions of COVID-19 than natives (*differential effects via disease susceptibility*)?

RQ3: Are immigrants more likely to seek or receive healthcare at a later stage in their disease than natives (*differential effects via individual or structural response*)?

RQ4: How do the above-mentioned mechanisms interact to increase risks of COVID-19 mortality among different immigrant groups and compared to natives?

METHODS AND ANALYSIS

Study population and study period

The study population includes all adults (≥ 18 years of age) registered in Sweden in the year before the start of the pandemic (January 31st 2019) and individuals who immigrated to Sweden or turned 18 years of age during the follow-up.

Our analyses will primarily cover the period from January 31st, 2020 (when the first confirmed case of COVID-19 was registered in Sweden) until December 31st, 2022, with updates depending on the progression of the pandemic. In order to evaluate the specific effect of the pandemic on immigrant-native health inequalities, we will systematically estimate differences in all-cause mortality the year before the pandemic.

We will categorize the study period into two stages. The first period (from January 31st to December 31st, 2020) reflects the period of time from the first of the first infection in Sweden until the start of the vaccination period (from January 1st, 2021, the first date of data for vaccinations in Sweden). The later period will be divided considering different timing in access to vaccination by age and risk groups.

Data

We will use multiple national registers linked through unique identification numbers[22]:

- Health registers, namely the *Cause of Death*[23], *Intensive Care (SIR), National Patient* (IPR),[24] *Prescription Drug,*[25] *SmiNet* and *National Vaccination Registers*, with data on deaths, intensive care unit (ICU) visits, inpatient hospitalizations and outpatient visits, drug prescriptions and dispensations (dates, diagnoses for all), COVID-19 PCR test results, and individual vaccination dates as well as sex and region for aggregated vaccination data, respectively;
- Socioeconomic registers, including the *Longitudinal Integration Database for Health Insurance and Labor Market Studies* (LISA) and *Longitudinal Database for Integration Studies* (STATIV), with annual data on socioeconomic indicators, social insurance benefits, municipality/county of residence, as well as immigrant-specific factors including country of birth, year of arrival in Sweden, and reason for migration;

For peer review only

- The *Multigenerational Register*,[26] to identify biological/adoptive family units;
- The *Dwelling Register*, to identify housing unit members, housing type, and dwelling characteristics;
- The *Care and Services for the Elderly Register* (SOL), to identify individuals living in elderly care homes; and
- The *Geographic Register*, for administrative (SAMS) and social (DeSO) definitions of neighborhoods.

We will use information from the above registers from different years. We will use socio-economic information on the study population from 1990 (the year of establishment of LISA) to examine social patterns in underlying health status (from 2015) associated with severe COVID-19 (from 2019).

COVID-19-related variables

This project will consider various COVID-19 outcomes along the disease pathway:

PCR tests: We will use data on positive COVID-19 PCR test dates and results. Depending on availability, we will examine individual- or aggregate-level test data by sex and Swedish region.

Outpatient care: Data from the National Patient Register will be used to capture outpatient visits coded with emergency/updated COVID-19 ICD codes (U07.1, U07.2, and B34.2), including dates to measure first contact with healthcare services for COVID-19 symptoms.

Hospitalizations: Data from the National Patient Register will be used to capture hospitalizations coded with emergency/updated COVID-19 ICD codes (see *Outpatient care*), including dates of admission and release.

Intensive care unit visits: Data from the Intensive Care Register will be used to capture ICU admissions coded with emergency/updated COVID-19 ICD codes (see *Outpatient care*), including dates of admission and release.

Mortality: Using the Cause of Death Register, we will capture deaths due to COVID-19, with dates and immediate/underlying causes of death (see *Outpatient care* for ICD codes). Given that COVID-19 mortality refers exclusively to confirmed cases, we will consider sensitivity analyses including deaths with a recent positive test and other causes of death (e.g., respiratory problems) that may be indicative of underreported COVID-19 mortality.

Delayed access to care: In order to measure delays in healthcare access, we will calculate the time from testing positive for COVID-19 (see *PCR tests*) or first contact with healthcare services regarding COVID-19 symptoms (see *Outpatient care*) to hospitalization and ICU admission.

Immigrant-specific variables

All individuals born outside Sweden, comprising approximately 20% of the Swedish total population,[27] will be considered as immigrants. They will be categorized by country/region of birth depending on the corresponding sample size and ethical considerations regarding identifiability. Duration of residence (<5, 5-10, >10 years), age

For peer review only

at arrival (school age, young adulthood, older adulthood), intermarriage (native-native, native-immigrant, immigrant-native, immigrant-immigrant couples) and immigrant background (second generation) will be examined.

Other relevant health variables

In order to measure health five years prior to a COVID-19 event, we will combine information from several health registers. We consider to calculate, e.g., the Charlson Comorbidity Index. Specific health conditions associated with COVID-19 susceptibility will be identified in the international literature.

Demographic and social variables

Across all studies, we will consider sex and age. Given that age is one of the most important predictors of COVID-19 prognosis, we will stratify our analyses by age groups, based on the availability of data for each age group and clinically-meaningful patterns of COVID-19 prognosis across these age groups.

In order to investigate social factors that may affect the risk of exposure to infection for RQ1 and RQ4, we will assess variables at the *individual level*, including type of occupation (identifying high-exposure jobs using the O*NET online database[28]) and employment type (including self-employed); *household level*, including type of dwelling (houses, apartments, care homes), household size (individuals per square meter), number of working-age individuals (and their occupation) and presence of multiple generations in the household; and *neighborhood/residential level*, including geographic proximity to family and the workplace, neighborhood population density (residents per square kilometer) and immigrant density (proportion foreign-born residents).

The following variables will be carefully considered as confounders, moderators or mediators using directed acyclic graphs (DAGs) for all research questions: individual- and area-level labor and disposable income, educational attainment, employment status, marital/cohabitation status, type and size of dwelling, and place of residence. Individual-level information will be used to account for area-level composition, while area-level information will be used to socioeconomically characterize the neighborhood of residence.

Study design and statistical analysis

This project will implement longitudinal study designs to effectively capture the timing of specific exposures and outcomes.

For RQ1, we will study the association between social factors hypothesized to influence risks of exposure to the virus and outcomes that confirm infection while comparing immigrants and natives.

We will apply multilevel models with three levels of analysis (individual, household, and neighborhood) in order to estimate *specific* (effect of individual- and higher-level variables on individual outcomes) and *general* (effect of each level on the outcome)

For peer review only

contextual effects, as well as to test cross-level interactions. We will consider 'neighbourhoods' by administrative (SAMS) and demographic definitions (DeSO). We will also consider incorporating a fourth analytical level corresponding to the 21 Swedish regions, which are responsible for managing their own public health and healthcare responses to the pandemic beyond national recommendations.

We plan to implement a joint multilevel survival analysis[29] in order to censor information by other causes of death or emigration. If this cannot be implemented due to computational feasibility, we will run logistic hierarchical models. Individual variance will be interpreted using median odds ratios (MOR, for logistic models) or median hazard ratios (MHR, for survival models) to evaluate the relative contribution of each level of analysis to the outcome. We will then compare model specifications (departing from an empty model) to estimate the proportional change in variance (PCV) between them and evaluate the extent to which differences in the outcome are attributable to area-level or compositional differences. Data on immigrant country/region of origin will be included as an individual-level variable (fixed characteristic) with natives as a reference. We will conduct additive and multiplicative interaction analyses to evaluate heterogeneity of exposure variables and outcomes between immigrants and natives.

For RQ2, we will conduct two types of analyses. First, we will evaluate native-immigrant differences pre-COVID-19 general health and comorbidities, as well as specific pre-existing health conditions and cause-specific mortality (the latter based on the literature regarding conditions linked to worse COVID-19 prognosis, e.g., cardiovascular and respiratory diseases).[30] We will apply Cox regression models to estimate time to first COVID-19 hospitalization and death, using age as a time scale and right-censoring by alternative (non-COVID-19-related) causes of death and potential emigration. Second, we will use mediation analysis to evaluate the extent to which native-immigrant differences in COVID-19 mortality are explained by pre-existing conditions at baseline (i.e., at the start of the pandemic). Together, these two approaches will allow us to estimate whether immigrants have poorer COVID-19 prognoses due to greater risks of poor health or health conditions associated with COVID-19 susceptibility.

Given our specific interest in socially-patterned conditions, we will also consider associations between the aforementioned health conditions and various social factors. In turn, we will be able to disentangle the extent to which pre-existing conditions involved in native-immigrant COVID-19 mortality differences are explained by social inequalities between these groups.

In order to compare the results between the first and second sets of analyses, we will use duration of residence information to proxy potential underdiagnosis of pre-existing health conditions among those who died from COVID-19, as underdiagnosis is common among newly-arrived immigrants. We will also use consider immigrant generation to discuss susceptibility in relation to social conditions at birth, as well as potential intergenerational transmission of susceptibility.

For RQ3, we will apply Cox regression to first evaluate native-immigrant differences in healthcare delay by calculating time from first contact with healthcare services for COVID-19 symptoms (i.e., testing positive, outpatient care) and hospitalization or ICU

For peer review only - http://bmjopen.bmj.com/site/about/guidelines.xhtml

admission. Second, we will study native-immigrant differences in COVID-19-related ICU admissions and mortality following confirmed infection to assess if and how prognosis differs by immigrant status.

Across both sets of analyses, to evaluate differential *individual response* within immigrant groups, we will consider duration of residence, age at arrival and occurrence of intermarriage as proxies for information that is unavailable in registers, e.g., inadequate language proficiency or poor understanding of the healthcare system. To evaluate *structural response*, we plan to assess whether differences in healthcare delay are greater during the peak of each wave compared to before or after the peak, by resource availability (e.g., ICU capacity). Peaks will be identified using mortality data.

For this research question, it is particularly relevant to evaluate period effects by different stages of the pandemic as well as regional differences. The proportion of individuals vaccinated by age and country/region of origin will be considered at different stages of the vaccination process, as this may also influence mortality differences.

For this final question (RQ4), we will build upon the previous steps by examining the joint role of the three mechanisms in COVID-19 mortality. We will begin by conducting Poisson regression to estimate relative risks (RR) along the disease pathway, for hospitalization (i.e., overall and among positively tested) and mortality (i.e., overall, among positively tested and hospitalized). This will help to consider the severity of the disease and whether excess mortality reflects greater exposure or poorer prognosis. The analyses will be stratified by different pandemic stages, as well as by factors such as intermarriage, to better account for language barriers, and duration of residence, to consider potential underdiagnosis of pre-existing health conditions.

We will then apply a three-way decomposition approach to determine whether inequalities are driven by differential exposure or effect (via susceptibility and/or response). Marginal structural modelling will be used to estimate the direct effects of origin and indirect effects mediated by intermediate factors (positive tests, pre-existing conditions and time between first contact with healthcare services and hospitalization). The interaction approach will allow us to examine outcome heterogeneity in subgroups with similar levels of exposure and/or vulnerabilities. This will also reduce the bias of imperfectly measured intermediate outcomes.

The proposed studies relating to RQ4 will require important theoretical and methodological consideration, some based on evidence from our prior planned research. For example, we will discuss how some social factors (e.g., occupation) may be a common cause for different pathways (e.g., differential exposure to the virus and differential effects via disease susceptibility). The explicit identification of different paths will be important for specific policy targets,[20] while the discussion regarding social causation will improve our understanding of COVID-19 inequalities.

For peer review only

LIMITATIONS

Most of the limitations in this study relate to problems of data availability and quality. First, we lack information on undocumented immigrants. Although we can identify undocumented (i.e., without a personal identification number) immigrant deaths, individuals cannot be linked to register data (i.e., social variables). Similarly, we are not able to identify ethnicity beyond the country of birth. Second, the project will study COVID-19 outcomes along the disease pathway, each of which may be affected by different sources of bias. For example, due to positive reporting bias (i.e., greater likelihood of taking a test following symptoms) and low testing rates. We may supplement test data with outpatient, hospitalization or ICU data coded for past COVID-19 infections to proxy a positive test result. By jointly examining these outcomes, however, we can shed light on the mechanisms behind their individual sources of error. For instance, comparing COVID-19 testing and mortality data may reveal shortcomings in testing rates. A Swedish report found that immigrants from Africa had higher mortality than natives, but similar risks of positive tests,[8] suggesting limited testing among specific immigrant groups who may still experience high COVID-19 infection rates. By combining such findings with other immigrant-specific variables, we aim to disentangle the reasons behind these discrepancies. As an example, a shrinking 'testing-mortality' discrepancy with longer duration of residence could suggest that testing may be hindered by limited access or knowledge due to language barriers in early residence.

The study will offer a thorough empirical evaluation of how social conditions shape group risks in the context of a pandemic giving rise to native-immigrant inequalities in COVID-19 mortality.

For peer review only

Patient and public involvement

This project will not involve any patients or the public. However, we will attempt to communicate the findings of the study to the public through the mass media.

Ethics and dissemination

Although we handle personal information, individual consent is not required since the information is anonymised by the agencies responsible for data protection, and none of the records will have personal identifiers attached. Statistics Sweden may also aggregate some information (such as country of birth) to ensure that individuals cannot be identified in relation to other variables (e.g., education or place of residence). Nevertheless, since our dataset contains individual-level information and thus requires special provisions for its use, we applied for all necessary ethical permissions from the National Ethical Review Board to cover the specific research questions addressed by this project (Dnr 2022-0048-01).

Author's contribution

SPJ conceived the study. HH, SA, ED, MR, EM and SVK give critical feedback. SJ drafted the manuscript with help of HH. SJ and ED designed the visualization of the theoretical framework. HH, SA, ED, MR, EM and SVK revised the manuscript. SPJ is the guarantor.

Funding statement

This work was supported by the Swedish Council for Health, Working Life, and Social welfare (FORTE) Grant number 2021-00271. In addition, SPJ acknowledges funding from the Swedish Council for Health, Working Life, and Social welfare (FORTE) Grant number 2016–07128. SVK acknowledges funding from a NRS Senior Clinical Fellowship (SCAF/15/02), the Medical Research Council (MC_UU_00022/2) and the Scottish Government Chief Scientist Office (SPHSU17).

Funders of the study had no involvement in study design and will not be involved in any parts of the study, including data collection, data analysis, data interpretation, or writing of the report.

Competing Interests Statement: None

For peer review only

References

1. WHO. COVID-19 weekly epidemiological update, 18 May 2021. 2021; https://www.who.int/emergencies/diseases/novel-coronavirus-2019/situation-reports. Accessed 23-05, 2021.
2. Pan D, Sze S, Minhas JS, et al. The impact of ethnicity on clinical outcomes in COVID-19: A systematic review. *EClinicalMedicine.* 2020;3(23):100404.
3. Niedzwiedz C, O'Donnell C, Dinesh JB, et al. Ethnic and socioeconomic differences in SARS-CoV-2 infection: prospective cohort study using UK Biobank. *BMC medicine.* 2020;18(160).
4. England PH. *COVID-19: review of disparities in risks and outcomes.* London: Public Health England;2020.
5. Sze S, Pan D, Nevill CR, et al. Ethnicity and clinical outcomes in COVID-19: a systematic review and meta-analysis. *EClinicalMedicine.* 2020;29:100630.
6. Hayward SE, Deal A, Cheng C, et al. Clinical outcomes and risk factors for COVID-19 among migrant populations in high-income countries: a systematic review. *Journal of Migration and Health.* 2021(3):100041.
7. Rostila M, Cederström A, Wallace M, Brandén M, Malmberg B. Disparities in COVID-19 deaths by country of birth in Stockholm, Sweden: A total population based cohort study. *AJE.* 2020.
8. Folkhälsomyndigheten. *Utrikesfödda och covid-19 Konstaterade fall, IVA-vård och avlidna bland utrikesfödda i Sverige 13 mars 2020 –15 februari 2021.* 2021.
9. Diaz E, Norredam M, Aradhya S, et al. Situational Brief: Migration and Covid-19 in Scandinavian Countries. Lancet Migration https://www.migrationandhealth.org/migration-covid19-briefs 2020.
10. Aradhya S, Brandén M, Drefahl S, et al. Lack of acculturation does not explain excess COVID-19 mortality among immigrants. A population-based cohort study. Stockholm Research Reports in Demography. Preprint. https://doi.org/10.17045/sthlmuni.13110365.v2 2020.
11. Honkaniemi H, Bacchus-Hertzman J, Frittzell J, Rostila M. Mortality by country of birth in the Nordic countries –a systematic review of the literature. *BMC Public Health.* 2017;17:511.
12. Todd A, Bambra C. Learning from past mistakes? The COVID-19 vaccine and the inverse equity hypothesis *European Journal of Public Health.* 2021;31(1):2.
13. Diderichsen F. How did Sweden fail the pandemic? *International Journal of Health Services.* 2021.
14. Patel AP, Paranjpe MD, Kathiresan NP, Rivas MA, Khera AV. Race, socioeconomic deprivation, and hospitalization for COVID-19 in English participants of a national biobank. *Int J Equity Health* 2020;19(114).
15. Dahlgren G, Whitehead M. *European strategies for tackling social inequities in health: levelling up, Part 2.* Copenhagen: WHO Regional Office for Europe;2007.
16. Gluckman P, Hanson M. *Developmental Origins of Health and Disease.* Cambridge: Cambridge University Press; 2006.
17. Kuh D, Ben-Shlomo Y, Hallqvist J, Power C. Life course epidemiology. *J Epidemiol Community Health.* 2003;57:778-783.
18. Diderichsen F, Hallqvist J, Whitehead M. Differential vulnerability and susceptibility: how to make use of recent development in our understanding of mediation and interaction to tackle health inequalities. *Int J Epidemiol.* 2019;1(48(1)):268-274.
19. Burström B, Tao W. Social determinants of health and inequalities in COVID-19. *Eur J Public Health.* 2020;30(4):617–618.
20. Katikireddi SV, Lal S, Carrol ED, et al. Unequal impact of the COVID-19 crisis on minority ethnic groups: a framework for understanding and addressing inequalities. *J Epidemiol Community Health.* 2021.
21. Loi M, Del Savio L, Stupka E. Social epigenetics and equality of opportunity. *Public Health Ethics.* 2013;6(2):142-153.

For peer review only

22. Ludvigsson JF, Otterblad-Olausson P, Pettersson BU, Ekbom A. The Swedish personal identity number: possibilities and pitfalls in healthcare and medical research. *Eur J Epidemiol.* 2009;24:659–667.
23. Brooke HL, Talbäck M, Hörnblad J, et al. The Swedish cause of death register. *Eur J Epidemiol.* 2017;32(9):765-773.
24. Ludvigsson JF, Andersson E, Ekbom A, et al. External review and validation of the Swedish national inpatient register. *BMC Public Health* 2011;11(450).
25. Wallerstedt SM, Wettermark B, Hoffmann M. The first decade with the Swedish Prescribed Drug Register - A systematic review of the output in the scientific literature. *Basic Clin Pharmacol Toxicol.* 2016;119(5):464-469.
26. Ekbom A. The Swedish Multi-generation Register. *Methods Mol Biol.* 2011;675:215-220.
27. Foreign-born by country of birth, sex and year of immigration 31 December 2020. Retrieved from Stockholm. 2021; https://www.scb.se/en/finding-statistics/statistics-by-subject-area/population/population-composition/population-statistics/.
28. O*NET onlinedatabase. https://www.onetonline.org/find/descriptor/result/4.C.2.c.1.b. Accessed 02-02, 2020.
29. Brilleman SL, Crowther MJ, Moreno-Betancur M, et al. Joint longitudinal and time-to-event models for multilevel hierarchical data. *Stat Methods Med Res.* 2019;28(12):3502-3515.
30. Clark A, Jit M, Warren-Gash C, et al. Global, regional, and national estimates of the population at increased risk of severe COVID-19 due to underlying health conditions in 2020: a modelling study. *Lancet Glob Health.* 2020;8(8):e1003-e1017.

For peer review only

Figure 1. Theoretical framework to investigate COVID-19 mortality among immigrants from a Social Determinants of Health perspective.

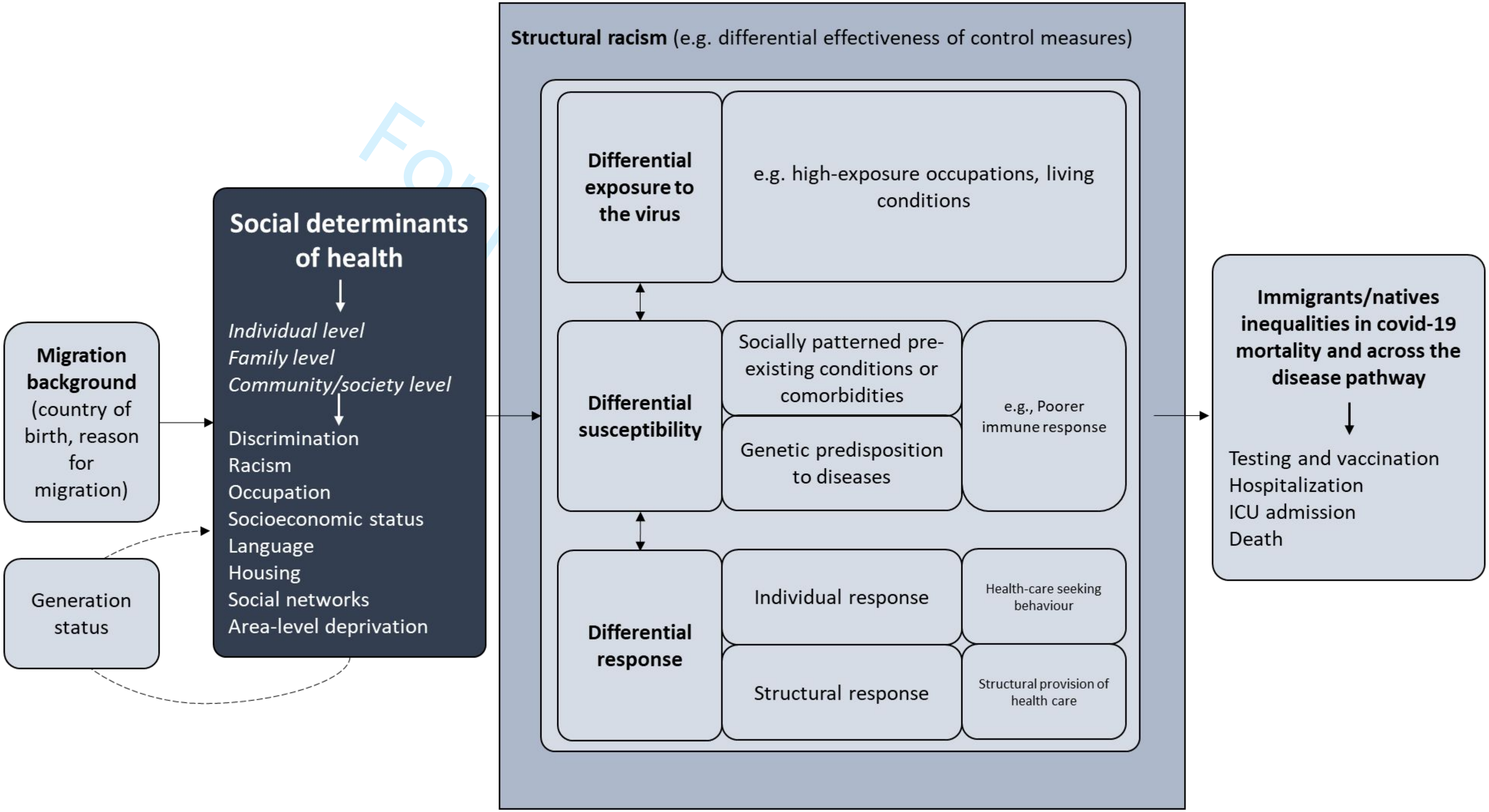

BMJ Open

Explaining COVID-19 mortality among immigrants in Sweden from a social determinants of health perspective (COVIS): protocol for a national register-based observational study

Journal:	*BMJ Open*
Manuscript ID	bmjopen-2022-070670.R1
Article Type:	Protocol
Date Submitted by the Author:	04-Apr-2023
Complete List of Authors:	Juárez , Sol Pia; Stockholm University, Department of Public Health Sciences; Centre for Health Equity Studies, Stockholm University/Karolinska Institute<br>Honkaniemi, Helena ; Stockholm University,<br>Aradhya, Siddartha ; Stockholm University,<br>Debiasi, Enrico; Stockholms Universitet, Department of Public Health Sciences<br>Katikireddi, Srinivasa; University of Glasgow, MRC/CSO Social & Public Health Sciences Unit<br>Cederström, Agneta; Stockholm University, Department of Public Health Sciences<br>Mussino, Eleonora; Stockholms Universitet, Stockholm University Demography Unit<br>Rostila, Mikael ; Stockholm University, Department of Public Health Sciences
**Primary Subject Heading**:	Public health
Secondary Subject Heading:	Public health
Keywords:	COVID-19, INFECTIOUS DISEASES, SOCIAL MEDICINE

SCHOLARONE™
Manuscripts

Explaining COVID-19 mortality among immigrants in Sweden from a social determinants of health perspective (COVIS): protocol for a national register-based observational study

Sol P. Juárez[1,2*] sol.juarez@su.se, ORCID iD: 0000-0001-9086-7588

Helena Honkaniemi[1,2] helena.honkaniemi@su.se, ORCID iD: 0000-0003-0800-0892

Siddartha Aradhya[3] siddartha.aradhya@sociology.su.se, ORCID iD: 0000-0003-3748-6270

Enrico Debiasi[1,2] enrico.debiasi@su.se, ORCID iD:0000-0001-5772-5510

Srinivasa Vittal Katikireddi[4] vittal.katikireddi@glasgow.ac.uk, ORCID iD: 0000-0001-6593-9092

Agneta Cederström[1,2] agneta.cederström@su.se, ORCID Id: 0000-0001-8866-7608

Eleonora Mussino[3] eleonora.mussino@sociology.su.se, ORCID iD: 0000-0002-5311-4277

Mikael Rostila[1,2] mikael.rostila@su.se, ORCID iD: 0000-0002-6973-0381

[1] Department of Public Health Sciences, Stockholm University, Stockholm, Sweden.

[2] Centre for Health Equity Studies, Stockholm University/Karolinska Institutet, Stockholm, Sweden.

[3] Demographic Unit (SUDA), Department of Sociology, Stockholm University, Stockholm, Sweden.

[4] Institute of Health & Wellbeing, University of Glasgow, Glasgow, UK

[*]Correspondence to:

Sol P. Juárez

Department of Public Health Sciences, Stockholm University, SE-106 91, Stockholm, Sweden

sol.juarez@su.se

Word count: 4,000(up to 4000)
Abstract: 250(up to 300)
Illustrations: 1
References: 31

For peer review only

For peer review only - http://bmjopen.bmj.com/site/about/guidelines.xhtml

Abstract

Introduction: Adopting a social determinants of health perspective, this project aims to study how disproportionate COVID-19 mortality among immigrants in Sweden is associated with social factors operating through *differential exposure* to the virus (e.g., by being more likely to work in high-exposure occupations) and *differential effects* of infection arising from socially-patterned, pre-existing health conditions, differential healthcare seeking and inequitable healthcare provision.

Methods and analysis: This observational study will use health (e.g., hospitalizations, deaths) and sociodemographic information (e.g., occupation, income, social benefits) from Swedish national registers linked using unique identity numbers. The study population includes all adults registered in Sweden in the year before the start of the pandemic (2019), as well as individuals who immigrated to Sweden or turned 18 years of age after the start of the pandemic (2020). Our analyses will primarily cover the period from January 31st, 2020 until December 31st, 2022, with updates depending on the progression of the pandemic. We will evaluate COVID-19 mortality differences between foreign- and Swedish-born individuals by examining each mechanism (differential exposure and effects) separately, while considering potential effect modification by country of birth and socioeconomic factors. Planned statistical modelling techniques include mediation analyses, multilevel models, Poisson regression and event history analyses.

Ethics and dissemination: This project has been granted all necessary ethical permissions from the National Ethical Review Board (Dnr 2022-0048-01) for accessing and analysing de-identified data. The final outputs will primarily be disseminated as scientific articles published in open-access peer-reviewed international journals, as well as press releases and policy briefs.

For peer review only

STRENGTHS AND LIMITATIONS OF THIS STUDY

- The study will offer a thorough empirical evaluation of how social conditions shape group risks in the context of a pandemic giving rise to native-immigrant inequalities in COVID-19 mortality.
- The study will offer a comprehensive understanding of native-immigrant inequalities across the COVID-19 disease pathway (i.e., positive test, hospitalization, intensive care unit admission and death).
- This study will be restricted to social information available in administrative registers, therefore it will not be able to account for self-reported racism and discrimination.
- Analyses will not be able to include undocumented immigrants, a highly vulnerable population.

For peer review only

For peer review only - http://bmjopen.bmj.com/site/about/guidelines.xhtml

INTRODUCTION

The severe acute respiratory syndrome coronavirus 2 (SARS-CoV-2) and its related disease (COVID-19) represent an unprecedented public health concern, with 760 million cases and 6.8 million deaths internationally as of March 2023.[1] Since the early stages of the pandemic, immigrants and ethnic minorities have been disproportionately affected by COVID-19. Notably, evidence from the UK and US has found that ethnic minorities are not only at a higher risk of being infected by SARS-CoV-2, but also of developing severe complications and dying from COVID-19, relative to majority ethnic groups.[2-5] A systematic review and meta-analysis has confirmed this pattern for immigrants in most high-income countries.[6]

Sweden has been no exception. Despite its generous immigrant integration approaches and guarantee of equal access to healthcare and social protection for all documented immigrants, immigrants from low- to high-income countries have exhibited excess COVID-19 mortality in Sweden.[7] However, some notable differences have been found by immigrants' region of origin. For example, while immigrants from Finland and Chile have shown moderately higher COVID-19 mortality risks than native-born Swedes, immigrants from other origins show relative risks approximately two (e.g., Iran, Iraq), five (e.g., Syria, Lebanon), and eight times (e.g., Somalia) higher than that of natives.[7] This observation contrasts sharply with the mortality advantage seen among immigrants relative to native Swedes for most other causes, both before and during the pandemic.[7-10]

In the international literature, multiple hypotheses have been put forth to explain the excess COVID-19 mortality observed among immigrants and ethnic minorities, two categories which are commonly examined together. In the early months of the pandemic, explanations often focused on biological arguments (including the role of genetic predispositions and vitamin D levels), with politicians claiming that the 'virus does not discriminate' by social background.[11] However, as the pandemic continued, hypotheses involving social factors gained more attention, especially as the empirical evidence began to contradict previous reductive biological arguments. For one, excess COVID-19 mortality has been observed across a wide range of ethnic minority groups, calling into question the role of unique biological drivers.[5] Furthermore, COVID-19 has been found to have a disproportionately greater impact on lower socioeconomic groups,[12,13] illustrating the socially unequal effects of the pandemic.

While the research and political debate regarding the disproportionate impact of the pandemic on minorities is now generally discussed within a broader framework of social inequalities, empirical research on the *mechanisms* behind the observed inequalities is limited. A mechanistic understanding of the role of social inequalities in the pandemic is crucial for addressing the effects of the current crisis on already disadvantaged immigrant and ethnic minority groups, while equipping the public health field for potential future outbreaks.

For peer review only - http://bmjopen.bmj.com/site/about/guidelines.xhtml

Theoretical foundation: Social Determinants of Health (SDH)

This study is rooted in the *Social Determinants of Health* (SDH),[14] a theoretical framework that considers health to be a product of various social factors operating at different levels (individual, family, community and/or society) giving rise to social distributions of health and disease in the population. Although broad, the SDH framework lays the foundation for the study of health inequalities, focusing on the underlying social causes of manifested biological differences between groups.

The SDH framework becomes more informative in conjunction with other theoretical perspectives. For example, researchers should also account for the Developmental Origins of Health and Disease[15] and the life-course[16] perspectives on health, which recognize that poor health is not only the result of the immediate social circumstances of an individual or group, but also of the individual and structural conditions that are present at birth or that accumulate over the lifespan, for example socioeconomic deprivation or experiences of racism and discrimination. Within these frameworks, health inequalities can be conceptualized as the biological expression of unequal lives, which can persist within and across generations.

The above-mentioned frameworks lay the foundation for understanding how health inequalities are (re)produced by social factors operating through exposure and susceptibility.[17] This collective theory of health inequalities has been recently adapted to the context of the COVID-19 pandemic, both for the Swedish general population[12,18] and for ethnic minority groups internationally.[19] In this study, we will further adapt the theory to specifically consider inequalities in COVID-19 mortality among immigrants in Sweden.

Our theoretical expectation is that inequalities in COVID-19 mortality could arise from social factors operating through various points in the disease pathway (**Figure 1**). First, social factors may result in *differential exposure* to the virus, i.e., modifying the risk of being infected with SARS-CoV-2 in the first place. Social factors may also influence the development of the disease once the infection has occurred, with *differential effects* due to *disease susceptibility* (i.e., socially patterned pre-existing health), as well as *individual* (i.e., healthcare seeking) and *structural* (i.e., healthcare provision) *responses*. These mechanisms, independently or in combination with one another, may therefore lead to differential risks of COVID-19 mortality.

More specifically, *differential exposure* to the virus[12,19] refers to how social conditions can place immigrants at greater risk of SARS-CoV-2 infection. For example, immigrants may be at risk by participating in frontline jobs or living in crowded households and densely populated or economically-deprived areas with fewer opportunities to maintain a safe physical distance (e.g., in neighborhoods with small local stores and recreational facilities, fewer transport options for commuting, and less access to outdoor space).

Differential effects via *disease susceptibility*[12,18] (also referred to as *vulnerability*[19]) relates to the biological capacity to fight against the virus once the infection has taken place. Immigrants may be at greater risk of mortality due to COVID-19 if they have more pre-existing health conditions or comorbidities, poorer nutritional status or immune

For peer review only - http://bmjopen.bmj.com/site/about/guidelines.xhtml

response than native Swedes.[19] The concept of susceptibility has traditionally been used to refer to a genetic predisposition to certain diseases, which immediately removes from the debate the topic of inequalities (or inequities, i.e., unfair and avoidable differences) to focus on *fixed* group-level variations. However, recent developments in epigenetics highlight the role of environmental conditions (including social inequalities) in shaping the expression of our genes and predisposing certain individuals and groups to poor health.[20] Such research allows us to situate the concept of susceptibility in the context of social inequalities.

Inequalities might also arise from *differential effects* of infection via *individual and structural responses*, that is, the inclination and capacity to seek and provide necessary healthcare. *Individual* inclination to seek care may depend on one's trust in the healthcare system (depending on e.g., prior experiences of discrimination), while one's capacity may be dependent on the knowledge required to navigate the system (depending on e.g., language proficiency and health literacy). Furthermore, individual responses can to a large extent be conditioned by *structural* responses, or the capacity of the healthcare system to adapt to the demands of the pandemic while maintaining an equitable response across all social groups. Unlike disease susceptibility, which would be expected to remain stable throughout the course of the pandemic, structural response capacity may evolve and improve over time as the healthcare system adapts to the pandemic and more long-term solutions are introduced, such as the vaccination campaign. Yet, it is important to acknowledge the potential for continued inequalities in care provision rooted in underlying structural racism.

The above-mentioned mechanisms are not mutually exclusive.[19] Most adverse social factors are likely to have a joint effect on COVID-19 outcomes by operating across multiple mechanisms, not only resulting in an additive response on health but a multiplicative burden. For example, a greater risk of *exposure* to the virus in certain occupations might be exacerbated among individuals with high disease susceptibility who experience delayed or inadequate access to health care (i.e., *differential effects*), resulting in more severe COVID-19 outcomes. In fact, given that social inequalities are implicated in all these mechanisms, it is actually expected that *differential exposure* to the virus and *differential effects* of infection would be highly correlated.

Finally, it is important to acknowledge the potential for *differential effectiveness of control measures*, or the varied implementation and impact of regional or national public health strategies to tackle COVID-19.[19] By moderating the above-mentioned mechanisms, these control measures may have altered COVID-19 inequalities between immigrants and natives. Uniform control strategies affecting the total population (e.g., lockdowns) may have reduced immigrant-native inequalities by minimizing overall exposure to the virus, while less restrictive control measures (e.g., work-from-home recommendations) may have selectively protected individuals with the capacity to follow measures, including individuals in non-service sectors. Information about control measures may have also been differentially accessible to natives and immigrants due to e.g., linguistic obstacles and lack of appropriate communication channels. Overall, the design and implementation of these control measures appears to have been shaped by pre-existing forms of institutional bias, including structural racism. Although we do not aim to estimate the

For peer review only - http://bmjopen.bmj.com/site/about/guidelines.xhtml

differential impact of control measures in this study, we will take them into consideration when discussing the results.

Aim and research questions

The purpose of this project is to study how elevated COVID-19 mortality risks among immigrants in Sweden is associated with social determinants acting through *differential exposure* to infection (e.g., being more likely to work in high-exposure occupations); and *differential effects* of infection resulting from socially-patterned health conditions (i.e., *disease susceptibility*) as well as social discrepancies in individual healthcare seeking and/or structural provision of healthcare (i.e., *individual* or *structural response*).

Research question (RQ) 1: Are immigrants more likely to be exposed to SARS-CoV-2 infection than natives (*differential exposure to the virus*)?

RQ2: Are immigrants more likely to have socially-patterned health conditions that predispose them to developing more severe versions of COVID-19 than natives (*differential effects via disease susceptibility*)?

RQ3: Are immigrants more likely to seek or receive healthcare at a later stage in their disease than natives (*differential effects via individual or structural response*)?

RQ4: How do the above-mentioned mechanisms interact to increase risks of COVID-19 mortality among different immigrant groups and compared to natives?

METHODS AND ANALYSIS

Study population and study period

The study population includes all adults (≥18 years old) registered in Sweden in the year before the start of the pandemic (January 31st, 2019) and individuals who immigrated to Sweden or turned 18 years old during follow-up.

Our analyses will primarily cover the period from January 31st, 2020 (when the first confirmed COVID-19 case was registered in Sweden) to December 31st, 2022, with updates depending on the development of the pandemic. In order to evaluate the specific effect of the pandemic on immigrant-native health inequalities, we will systematically estimate differences in all-cause mortality the year before the pandemic.

We will categorize the study period into two stages. The first period (from January 31st to December 31st, 2020) reflects the time from the first recorded infection in Sweden until the start of the vaccination period (or the first date of vaccination data in Sweden, i.e., January 1st, 2021). The later period will be divided by considering different phases of access to vaccination by age and risk groups.

Data

We will use multiple national registers linked by unique identification numbers:[21]

For peer review only - http://bmjopen.bmj.com/site/about/guidelines.xhtml

- Health registers, namely the *Cause of Death* (CDR),[22] *Intensive Care (SIR), National Patient* (NPR),[23] *Prescription Drug,*[24] *SmiNet* and *National Vaccination Registers*, with data on deaths, intensive care unit (ICU) visits, inpatient hospitalizations and outpatient visits, and drug prescriptions and dispensations (with dates, diagnoses for all), as well as COVID-19 PCR test results, individual vaccination dates, and sex and region for aggregated vaccination data, respectively;
- Socioeconomic registers, including the *Longitudinal Integrated Database for Health Insurance and Labor Market Studies* (LISA) and *Longitudinal Database for Integration Studies*, with annual data on socioeconomic indicators, social insurance benefits, municipality/county of residence, as well as immigrant-specific factors including country of birth, year of arrival in Sweden, and reason for migration;
- The *Multigenerational Register*,[25] to identify biological/adoptive family units;
- The *Dwelling Register*, to identify housing unit members, housing type, and dwelling characteristics;
- The *Care and Services for the Elderly Register*, to identify individuals living in elderly care homes; and
- The *Geographic Register*, for administrative (Municipality, County and Small Area Market Statistics or SAMS) and social (Demographic Statistical Areas or DeSO) definitions of neighborhoods.

We will use information from the above registers over different years. We will use socioeconomic information on the study population from 1990 (the year of establishment of LISA) to examine social patterns in underlying health status (from 2015) associated with severe COVID-19 (from 2019).

COVID-19-related variables

This project will consider various COVID-19 outcomes along the disease pathway:

PCR tests: We will use data on positive COVID-19 PCR test dates and results. Depending on availability, we will examine individual- or aggregate-level test data by sex and Swedish region.

Outpatient care: Data from the NPR will be used to capture outpatient visits coded with emergency/updated COVID-19 ICD codes: B34.2, U07.1 (diagnosed with laboratory tests), U07.2 (clinically diagnosed when laboratory tests are unreliable or unavailable); and dates to measure first contact with healthcare services for COVID-19 symptoms.

Hospitalizations: Data from the NPR will be used to capture hospitalizations coded with emergency/updated COVID-19 ICD codes, including dates of admission and release.

ICU admissions: Data from the SIR will be used to capture ICU admissions coded with emergency/updated COVID-19 ICD codes, including dates of admission and release.

Mortality: Using the CDR, we will capture deaths due to COVID-19, with dates and immediate/underlying causes of death by ICD codes. Given that COVID-19 mortality refers exclusively to confirmed cases, we will also evaluate excess mortality (i.e., mortality levels exceeding those in the same month in pre-pandemic years) and consider

For peer review only - http://bmjopen.bmj.com/site/about/guidelines.xhtml

sensitivity analyses including deaths with a recent positive test but other cause of death (e.g., respiratory problems) that may be indicative of underreported COVID-19 mortality.

Delayed access or under-utilization of care: This will be proxied by different outcomes, including COVID-19 mortality outside hospitals; and time elapsed between testing/treatment phases. The latter includes time from testing positive for COVID-19 (see *PCR tests*) or first contact with healthcare services regarding COVID-19 symptoms (see *Outpatient care*) to hospitalization; from COVID-19-related hospitalization to ICU admission; and from ICU admission to death.

Immigrant-specific variables

All individuals born outside Sweden (approximately 20% of the Swedish population[26]) will be considered immigrants. They will be categorized by country/region of birth depending on the corresponding sample size and ethical considerations regarding identifiability. Duration of residence (<5, 5-10, >10 years), age at arrival (school-aged, young adult, older adult), intermarriage (native-native, native-immigrant, immigrant-native, immigrant-immigrant couples) and immigrant background (second generation) will be examined.

Other relevant health variables

In order to measure health five years prior to a COVID-19 event, we will combine information from several health registers. We will primarily examine specific health conditions that have been shown to be associated with COVID-19 severity.[27] This includes chronic kidney disease (ICD-10 code: N18), diabetes (E08-E13), cardiovascular diseases (E78, G45-G46, I10-I13, I20-I26, I63-I66, I80-I82), neurological problems (G20, G21, G30, G35-G37), chronic respiratory diseases (J40-J47), tuberculosis (A15-A19), HIV (B20-B24), chronic liver disease (K70-K77), cancer (C00-C96) and thalassemia or sickle-cell disorders (D56-D59). In addition, for the older adult population (65+ years old) we will also calculate more general comorbidity measures, e.g., the Charlson Comorbidity Index.

Demographic and social variables

Across all studies, we will consider sex and age. Given that age is one of the most important predictors of COVID-19 prognosis, we will stratify our analyses by age groups, based on the availability of data for each age group and clinically-meaningful patterns of COVID-19 prognosis across these age groups.

In order to investigate social factors that may affect the risk of exposure to infection for RQ1 and RQ4, we will assess variables at the *individual level*, including type of occupation (identifying high-exposure jobs using the O*NET online database[28]) and employment type (including self-employed); *household level*, including type of dwelling (houses, apartments, care homes), household size (individuals per square meter), number of working-age individuals (and their occupation), and presence of multiple generations in the household; and *neighborhood/residential level*, including geographic proximity to family and the workplace, neighborhood population density (residents per square kilometer), and immigrant density (proportion foreign-born residents).

For peer review only - http://bmjopen.bmj.com/site/about/guidelines.xhtml

The following variables will be carefully considered as confounders, moderators or mediators using directed acyclic graphs for all research questions: individual- and area-level labor and disposable income, educational attainment, employment status, marital/cohabitation status, type and size of dwelling, and place of residence. Individual-level information will be used to account for area-level composition, while area-level information will be used to socioeconomically characterize the neighborhood of residence.

Study design and statistical analysis

This project will implement longitudinal study designs to effectively capture the timing of specific exposures and outcomes.

For RQ1 (*differential exposure to the virus*), we will compare the association of social factors hypothesized to influence risks of exposure and outcomes that confirm infection between immigrants and natives. We will apply multilevel models with three levels of analysis (individual, household, neighborhood) in order to estimate specific (effect of individual- and higher-level variables on individual outcomes) and general (effect of each level on the outcome) contextual effects, as well as to test cross-level interactions. We will also consider incorporating a fourth analytical level corresponding to the 21 Swedish regions, which are responsible for managing their own public health and healthcare responses to the pandemic beyond national recommendations.

We plan to implement a joint multilevel survival analysis in order to censor information by other causes of death or emigration.[29] In case of a lack of computational feasibility, we will run logistic hierarchical models. Individual variance will be interpreted using median hazard ratios (survival models) or median odds ratios (logistic models) to evaluate the relative contribution of each level of analysis to the outcome. We will then compare model specifications to an empty model to estimate the proportional change in variance and evaluate the extent to which differences in the outcome are attributable to area-level or compositional differences. Data on immigrant country/region of origin will be included as an individual-level variable (fixed characteristic) with native references. We will conduct additive and multiplicative interaction analyses to evaluate heterogeneity of exposure variables and outcomes between immigrants and natives.

For this question, two main concerns include positive reporting bias (i.e., greater likelihood of taking a test following symptoms) and low testing rates among immigrants. A Swedish report found that immigrants from Africa had higher mortality than natives, but similar risks of positive tests,[30] suggesting limited testing among specific immigrant groups who may still experience high COVID-19 infection rates. In order to overcome this challenge, we plan to supplement test data with outpatient, hospitalization or ICU data coded for past COVID-19 infections to proxy a positive test result.

For RQ2 (*differential effects via disease susceptibility*), we will conduct two types of analyses. First, we will apply Cox regression to estimate time to first COVID-19 hospitalization and death, using age as a time scale and right-censoring by alternative

For peer review only - http://bmjopen.bmj.com/site/about/guidelines.xhtml

(non-COVID-19-related) causes of death and emigration. Second, we will use mediation analyses to evaluate the extent to which native-immigrant differences in COVID-19 mortality are explained by pre-existing conditions at baseline (i.e., at the start of the pandemic).

Given our interest in socially-patterned conditions, we will also examine associations between the aforementioned health conditions and various social factors. In turn, we will be able to disentangle the extent to which pre-existing conditions involved in native-immigrant COVID-19 mortality differences are explained by social inequalities between these groups.

Underdiagnosis of pre-existing health conditions is one of the main challenges associated with this question. We thus plan to run sub-group analyses restricting the population to immigrants residing in Sweden for more than 10 years, since they may be more comparable to natives in terms of their healthcare-seeking and diagnosis rates.

For RQ3 (*differential effects via disease response*), we will use various approaches to determine whether immigrants have received less healthcare or at a later stage of the COVID-19 disease than natives. These include Poisson regression models to estimate relative risks of excess mortality between immigrants and native Swedes outside hospitals (i.e., to capture unmet health needs) compared to before the pandemic. In addition, we will apply event history analyses with multiple time-scales[31] to evaluate transition rates between stages (testing/no-testing, hospitalization/no-hospitalization, ICU admission/no-ICU admission, death/no-death) while including age and calendar time (i.e., pandemic waves). This approach will allow us to determine not only differential time elapsed between testing/treatment stages but also differential trajectories of testing/treatment between immigrants and natives. To disentangle the potentially confounding influence of differential susceptibility to severe disease (and potentially more rapid disease progression, see RQ2), we will control for the presence of pre-existing conditions.

Sub-group analyses will be conducted to distinguish *differential individual* and *structural response* mechanisms underlying native-immigrant care timelines. For *individual response* mechanisms, we will distinguish immigrant groups by duration of residence, age at arrival, and occurrence of intermarriage to proxy individual obstacles to healthcare-seeking, e.g., language proficiency or institutional awareness. To evaluate *structural response* mechanisms, we plan to assess whether inequalities in healthcare delay differ by time (within and across waves) and regions, during which resource availability (e.g., ICU capacity) and other institutional factors (e.g., vaccination progress) may have variably affected healthcare delivery to specific groups.

One challenge for this question is the potential measurement error affecting the timing and type of treatment for COVID-19 disease. For one, uncaptured delays in testing, not just treatment, among immigrants may underestimate the true time elapsed between infection and treatment. However, with postponed testing, we would in fact expect a shorter time between positive test and hospitalization/ICU admission/death among immigrants than natives. Thus, whether natives or immigrants have a greater delay in

For peer review only - http://bmjopen.bmj.com/site/about/guidelines.xhtml

receiving care could explain the primary underlying mechanism of postponed testing versus treatment.

More broadly speaking, attempts to disentangle the mechanisms from one another (e.g., differential effects via disease susceptibility, individual response or structural response) may also be prone to bias. Although we plan to control for susceptibility through relevant pre-existing conditions, underdiagnosis may remain an issue (see RQ2). Similarly, it may be difficult to determine whether differential responses to the disease are due to individual healthcare-seeking behaviors or structural obstacles. We will thus rely on sub-group analyses to better understand these differing mechanisms, theorizing that more 'integrated' immigrants, e.g., those with longer residence or native partners, are less prone to underdiagnosis or healthcare-seeking obstacles, and thus more likely to be affected by structural obstacles such as inequitable resource allocation and healthcare delivery due to, e.g., structural racism.

Finally, for RQ4 (all mechanisms), we will compile an overview of important theoretical and methodological considerations based on evidence from the first three RQs. For example, we will discuss how some social factors (e.g., occupation) may be a common cause for different pathways (e.g., differential exposure and differential effects via disease susceptibility). The explicit identification of different paths will be important for specific policy targets,[19] while the discussion regarding social causation will improve our understanding of COVID-19 inequalities. We plan to summarize the empirical studies of this project by considering potential interventions that can help prevent inequalities among immigrants in future pandemics.

Studies are planned for 2023-2025.

Patient and public involvement

This project will not involve any patients or the public. However, we will attempt to communicate the findings of the study to the public through the mass media.

ETHICS AND DISSEMINATION

Although we handle personal information, individual consent is not required since the information is anonymised by the agencies responsible for data protection, and none of the records will have personal identifiers attached. Statistics Sweden may also aggregate some information (such as country of birth) to ensure that individuals cannot be identified in relation to other variables (e.g., education or place of residence). Nevertheless, since our dataset contains individual-level information and thus requires special provisions for its use, we applied for and were granted all necessary ethical permissions from the National Ethical Review Board to cover the specific research questions addressed by this project (Dnr 2022-0048-01).

For peer review only - http://bmjopen.bmj.com/site/about/guidelines.xhtml

The final outputs will primarily be disseminated as scientific articles published in open-access peer-reviewed international journals, as well as press releases and policy briefs.

DISCUSSION

Most of the limitations in this study relate to problems of data availability and quality. First, we lack information on undocumented immigrants. Although we can identify undocumented (i.e., without a personal identification number) immigrant deaths, individuals cannot be linked to register data (i.e., social variables). Similarly, we are not able to identify ethnicity beyond the country of origin. Second, the project will study COVID-19 outcomes along the disease pathway, each of which may be affected by different sources of bias. Although we are not able to evaluate the role of the Swedish public health strategies specifically, we acknowledge that the lack of lockdown mandates, for example, can make our results less generalizable to other contexts, yet open opportunities for collaboration with research groups from other national contexts. The fact that Sweden did not have strong mandates (i.e., lockdowns and face masks rules outside medical settings) will allow us to better examine the role of the different proposed mechanisms.

Despite these limitations, this study uses high-quality population registers that link multiple health and social outcomes before and after the start of the pandemic. The study will offer a thorough empirical evaluation of how social conditions shape group risks in the context of a public health crisis and can give rise to increased understanding of these mechanisms. Our findings will thus contribute knowledge to mitigating and potentially preventing such inequalities in future pandemics.

Contributors

SPJ conceived the study. HH, SA, ED, MR, EM, SVK and AC give critical feedback. SJ drafted the manuscript with help of HH. SJ and ED designed the visualization of the theoretical framework. HH, SA, ED, MR, EM, SVK, AC revised the manuscript. SPJ is the guarantor.

Funding

This work was supported by the Swedish Council for Health, Working Life, and Social welfare (FORTE) Grant number 2021-00271. In addition, SPJ acknowledges funding from the Swedish Council for Health, Working Life, and Social welfare (FORTE) Grant number 2016–07128. SVK acknowledges funding from a NRS Senior Clinical Fellowship (SCAF/15/02), the Medical Research Council (MC_UU_00022/2) and the Scottish Government Chief Scientist Office (SPHSU17). Funders of the study had no involvement in study design and will not be involved in any parts of the study, including data collection, data analysis, data interpretation, or writing of the report.

Competing interests

None.

Acknowledgements

For peer review only - http://bmjopen.bmj.com/site/about/guidelines.xhtml

We would like to thank Dr. Anders Ledberg for his valuable insights on this protocol.

For peer review only

References

1. WHO. COVID-19 weekly epidemiological update, 18 May 2021. 2021; https://www.who.int/emergencies/diseases/novel-coronavirus-2019/situation-reports. Accessed 23-05, 2021.
2. Pan D, Sze S, Minhas JS, et al. The impact of ethnicity on clinical outcomes in COVID-19: A systematic review. *EClinicalMedicine.* 2020;3(23):100404.
3. Niedzwiedz C, O'Donnell C, Dinesh JB, et al. Ethnic and socioeconomic differences in SARS-CoV-2 infection: prospective cohort study using UK Biobank. *BMC medicine.* 2020;18(160).
4. England PH. *COVID-19: review of disparities in risks and outcomes.* London: Public Health England;2020.
5. Sze S, Pan D, Nevill CR, et al. Ethnicity and clinical outcomes in COVID-19: a systematic review and meta-analysis. *EClinicalMedicine.* 2020;29:100630.
6. Hayward SE, Deal A, Cheng C, et al. Clinical outcomes and risk factors for COVID-19 among migrant populations in high-income countries: a systematic review. *Journal of Migration and Health.* 2021(3):100041.
7. Rostila M, Cederström A, Wallace M, Brandén M, Malmberg B. Disparities in COVID-19 deaths by country of birth in Stockholm, Sweden: A total population based cohort study. *AJE.* 2020.
8. Diaz E, Norredam M, Aradhya S, et al. Situational Brief: Migration and Covid-19 in Scandinavian Countries. Lancet Migration https://www.migrationandhealth.org/migration-covid19-briefs 2020.
9. Aradhya S, Brandén M, Drefahl S, et al. Lack of acculturation does not explain excess COVID-19 mortality among immigrants. A population-based cohort study. Stockholm Research Reports in Demography. Preprint. https://doi.org/10.17045/sthlmuni.13110365.v2 2020.
10. Honkaniemi H, Bacchus-Hertzman J, Fritzell J, Rostila M. Mortality by country of birth in the Nordic countries –a systematic review of the literature. *BMC Public Health.* 2017;17:511.
11. Todd A, Bambra C. Learning from past mistakes? The COVID-19 vaccine and the inverse equity hypothesis *European Journal of Public Health.* 2021;31(1):2.
12. Diderichsen F. How did Sweden fail the pandemic? *International Journal of Health Services.* 2021.
13. Patel AP, Paranjpe MD, Kathiresan NP, Rivas MA, Khera AV. Race, socioeconomic deprivation, and hospitalization for COVID-19 in English participants of a national biobank. *Int J Equity Health* 2020;19(114).
14. Dahlgren G, Whitehead M. *European strategies for tackling social inequities in health: levelling up, Part 2.* Copenhagen: WHO Regional Office for Europe;2007.
15. Gluckman P, Hanson M. *Developmental Origins of Health and Disease.* Cambridge: Cambridge University Press; 2006.
16. Kuh D, Ben-Shlomo Y, Hallqvist J, Power C. Life course epidemiology. *J Epidemiol Community Health.* 2003;57:778-783.
17. Diderichsen F, Hallqvist J, Whitehead M. Differential vulnerability and susceptibility: how to make use of recent development in our understanding of mediation and interaction to tackle health inequalities. *Int J Epidemiol.* 2019;1(48(1)):268-274.
18. Burström B, Tao W. Social determinants of health and inequalities in COVID-19. *Eur J Public Health.* 2020;30(4):617–618.
19. Katikireddi SV, Lal S, Carrol ED, et al. Unequal impact of the COVID-19 crisis on minority ethnic groups: a framework for understanding and addressing inequalities. *J Epidemiol Community Health.* 2021.
20. Loi M, Del Savio L, Stupka E. Social epigenetics and equality of opportunity. *Public Health Ethics.* 2013;6(2):142-153.

For peer review only - http://bmjopen.bmj.com/site/about/guidelines.xhtml

21. Ludvigsson JF, Otterblad-Olausson P, Pettersson BU, Ekbom A. The Swedish personal identity number: possibilities and pitfalls in healthcare and medical research. *Eur J Epidemiol.* 2009;24:659–667.
22. Brooke HL, Talbäck M, Hörnblad J, et al. The Swedish cause of death register. *Eur J Epidemiol.* 2017;32(9):765-773.
23. Ludvigsson JF, Andersson E, Ekbom A, et al. External review and validation of the Swedish national inpatient register. *BMC Public Health* 2011;11(450).
24. Wallerstedt SM, Wettermark B, Hoffmann M. The first decade with the Swedish Prescribed Drug Register - A systematic review of the output in the scientific literature. *Basic Clin Pharmacol Toxicol.* 2016;119(5):464-469.
25. Ekbom A. The Swedish Multi-generation Register. *Methods Mol Biol.* 2011;675:215-220.
26. Foreign-born by country of birth, sex and year of immigration 31 December 2020. Retrieved from Stockholm. 2021; https://www.scb.se/en/finding-statistics/statistics-by-subject-area/population/population-composition/population-statistics/.
27. Clark A, Jit M, Warren-Gash C, et al. Global, regional, and national estimates of the population at increased risk of severe COVID-19 due to underlying health conditions in 2020: a modelling study. *Lancet Glob Health.* 2020;8(8):e1003-e1017.
28. O*NET onlinedatabase. https://www.onetonline.org/find/descriptor/result/4.C.2.c.1.b. Accessed 02-02, 2020.
29. Brilleman SL, Crowther MJ, Moreno-Betancur M, et al. Joint longitudinal and time-to-event models for multilevel hierarchical data. *Stat Methods Med Res.* 2019;28(12):3502-3515.
30. Folkhälsomyndigheten. *Utrikesfödda och covid-19 Konstaterade fall, IVA-vård och avlidna bland utrikesfödda i Sverige 13 mars 2020 –15 februari 2021.* 2021.
31. Iacobelli S, Carstensen B. Multiple time scales in multi-state models. *Statist Med.* 2013(32):315–5327.

For peer review only - http://bmjopen.bmj.com/site/about/guidelines.xhtml

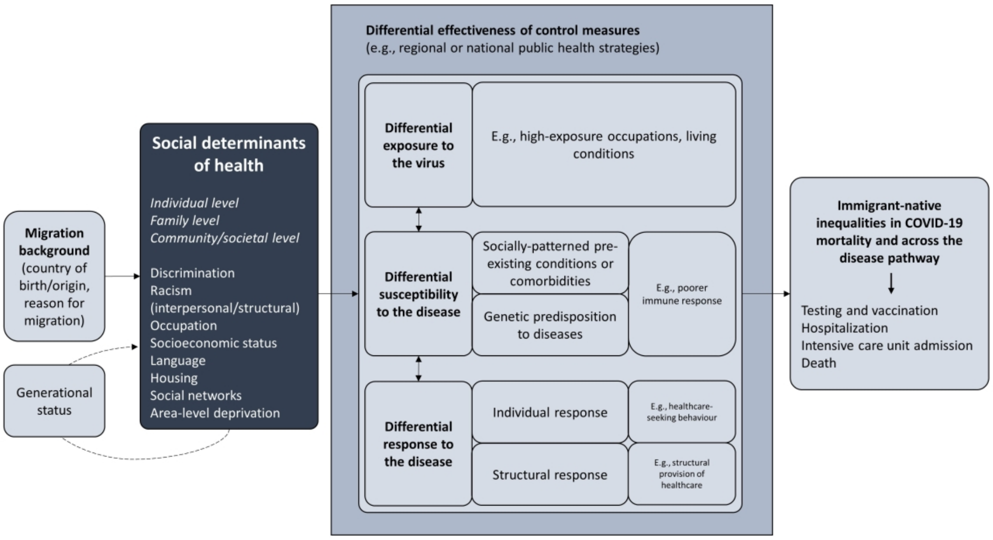

Figure 1

338x190mm (300 x 300 DPI)
